# *In Vivo* Effects of Cagaita (*Eugenia dysenterica*, DC.) Leaf Extracts on Diarrhea Treatment

**DOI:** 10.1155/2011/309390

**Published:** 2010-08-30

**Authors:** T. B. Lima, O. N. Silva, L. P. Silva, T. L. Rocha, M. F. Grossi-de-Sá, O. L. Franco, E. Leonardecz

**Affiliations:** ^1^Centro de Análises Proteômicas e Bioquímicas, Programa de Pós-Graduação em Ciências Genômicas e Biotecnologia, Universidade Católica de Brasília, SGAN Quadra 916, Modulo B, Avenue W5, 70 790-160 Brasília, DF, Brazil; ^2^Programa de Pós-graduação em Ciências Biológicas (Imunologia/Genética e biotecnologia), Universidade Federal de Juiz de Fora, 36036-330 Juiz de Fora, MG, Brazil; ^3^Embrapa Recursos Genéticos e Biotecnologia, Laboratório de Espectrometria de Massa e Laboratório de Interação Molecular Planta-Praga, PqEB, Avenue W5 Norte, 70770-900 Brasília-DF, Brazil; ^4^Universidade de Brasília, Campus Universitário de Planaltina Vila Nossa Senhora de Fátima, 73300-000 Brasília, DF, Brazil

## Abstract

*Eugenia dysenterica* is a plant typically found in the Cerrado biome and commonly used in popular medicine due to its pharmacological properties, which include antidiarrheal, skin healing, and antimicrobial activities. The effects of ethanolic extract, aqueous extract and infusion of *E. dysenterica* leaves on intestinal motility and antidiarrheal activity were evaluated using ricin oil-induced diarrhea in rats. At doses of 400 and 800 mg·Kg^−1^, the ethanolic extract decreased intestinal motility while the other extracts showed no significant effects. Moreover, serum levels of chloride, magnesium, and phosphorus were also measured in rats. Histopathologic and enzymatic analyses were also performed to investigate any toxic effect. Animals treated with infusion, ethanolic extract, ricin oil, and loperamide presented morphological alterations in the small intestine, such as mucosa lesion, epithelial layer damage, and partial loss and/or morphological change of villi. Furthermore, the liver showed congestion and hydropic degeneration. Serum levels of alanine aminotransferase increased significantly in all treatments, but none rose above reference values. In summary, our results suggest that compounds present in leaves of *E. dysenterica* may have therapeutic benefits on recovery from diarrhea despite their toxic effects.

## 1. Introduction

Diarrhea is responsible for deaths in adults and especially children all over the world, particularly in developing countries [1, 2]. In general, chronic diarrhea can develop from congenital problems, neoplastic syndrome, human immunodeficiency virus (HIV), adverse effects of medications, and, most commonly, is caused by gastrointestinal infections including viral/bacterial gastroenteritis [3, 4]. In general, patients with uncontrolled diarrhea are at increased risk of dehydration, electrolyte imbalance, skin breakdown, and fatigue. In many cases the treatments utilized are oral rehydration therapy (ORT) and pharmacological intervention, including antibiotics and antidiarrheal drugs, which cause a pronounced effect on gut motility, thus decreasing intestinal transit [5, 6]. Currently, the search for antidiarrheal therapies should include allopathic, homeopathic, and alternative medical approaches that have demonstrated their ability to reduce the duration and severity of diarrhea in children in developing countries [7]. 

In view of a number of disease properties including epidemiology, etiological agents, and changeable clinical manifestations depending on the region, different strategies have been applied to control diarrhea [8]. In order to reduce infections caused by bacterial pathogens, the Diarrheal Disease Control program created in 1978 by the World Health Organization (WHO) puts special emphasis on the use of traditional (folk) medicines in the control and management of diarrhea [9]. The regional Western Pacific Office of the WHO included diarrhea as a target disease [10]. Currently, screening of biological products and phytochemical extracts has been encouraged to obtain isolated plant biomolecules with antidiarrheal activity, including various plant families such as Liliaceae [11], Rubiaceae [12], Meliaceae [13], Fabaceae [14], Myrtaceae [15], and several others. 

*Eugenia dysenterica* DC., known as “cagaita”, is a fruit-bearing tree of the Myrtaceae family native to the Brazilian Cerrado (upland savannah). This plant is widely used by the population in the treatment of various diseases; tea from its leaves is used to treat diarrhea, diabetes, and jaundice; tea from its flowers is used to treat kidney and bladder infections; its fruit is used as a laxative [16]. Considering that cagaita leaf tea is the main way in which this plant is used as a popular alternative medicine in Latin America, the present study was conducted to evaluate the possible antidiarrheal effect of the leaves of *E. dysenterica*, analyzing antimotility and antisecretory actions. Furthermore, the possible toxic effects were evaluated in a rat model by histopathological analysis.

## 2. Material and Methods

### 2.1. Plant Material and Extract Preparation

Leaves from *E. dysenterica* were collected in the Cerrado biome, from an area of upland savannah surrounding Brasilia-DF, Brazil. Leaves were dried at 30°C in a circulating air oven. One hundred grams of leaf powder was subjected to sequential extraction using solvents of increased polarity to afford dry extracts of hexane 100% in a proportion of 3 : 1 (w/v), ethanol 100% in a proportion of 3 : 1 (w/v), and deionized water (1 : 1) to give hexanic, ethanolic, and aqueous extracts [16]. The solvent of ethanolic extract was evaporated under vacuum in a rotary-evaporator at 45°C and further stored at 24°C. Aqueous extract was lyophilized and stored at −20°C. The powdered leaves (100 g) were also macerated and extracted with deionized water for 24 hours at 40°C under 120 rpm agitation. After cooling, the resulting extract was filtered through 8 *μ*m filter paper and lyophilized. For bioactivity investigations, all extracts were soluble in distilled water. None difficulty in the extract solubilization was observed. Percentage yields of the hexanic, ethanolic, and aqueous extracts and of the infusion were, respectively, 8.78%, 22.1%, 22.4%, and 20.0% of dry weight.

### 2.2. Animals

Male adult Wistar rats (*Rattus norvegicus*) weighing 280–300 g were acquired at the Biotery of the Universidade Católica de Brasília. All animals were used after 1 week of acclimatization (temperature 23 ± 2°C humidity 60%) and had free access to water and food. The study was approved by the Animal Use Committee (CEUA) at the Institute of Biological Sciences, University of Brasilia. Project number 0177–07 was approved on 27.03.07 under the name “Activity of drugs in the presence or absence of nanocapsules and/or magnetic fluids in the treatment of several diseases in experimental animal models”. The experiments reported here comply with ethical procedures with investigated animals [17].

### 2.3. Assessment of Gastrointestinal Propulsion of Charcoal Meal

Evaluation of antidiarrheal effects of *E. dysenterica* was conducted according to the method described by Abdullahi et al. [18]. Forty rats were separated in five groups of eight animals. The positive control group received loperamide orally, at the dose of 2 mg·kg^−1^_, _and the negative group received 1 mL of water; test groups received the infusion or aqueous extract at doses of 800 mg·kg^−1^ or ethanolic extract at doses of 400 mg·kg^−1^ resuspended in 1 mL of water. This procedure was determined according to Sagar et al. [19], Mbagwu and Adeyemi [20], Ojewole et al. [21], Mujumdar et al. [22], and Afroz et al. [23] with minor modifications. Test animals were starved for 12 hours prior to the experiment, but consumed water *ad libitum*. Ten min later, animals from each group were fed on 1 mL of charcoal meal (3% suspension of deactivated charcoal in 0.5% aqueous methylcellulose). Thirty minutes after the administration of charcoal meal, the animals from each group were killed by using CO_2_, and intestine length (pyloric sphincter to caecum) was measured, as well as the distance along which the charcoal moved as a fraction of that length, in accordance with the procedure described by Nwafor et al. [11]. The mean value for each group was calculated and results obtained in the control and test groups were compared. Percentage inhibitions of intestinal motility were calculated by using the equation (negative control mean—treated (test) mean)/negative control mean × 100.

### 2.4. Evaluation of Serum Levels

Analyses of ionic absorption and acute and subchronic toxicity after ricin oil-induced diarrhea were conducted according to the method described by Sagar et al. [19] with some modifications. Rats were divided into six groups of eight animals; diarrhea was induced by administering 1 ml of ricin oil orally using an intragastric tube. Thirty minutes after the administration of ricin oil, the animals received the treatments. The positive control group received loperamide orally, at the dose of 2 mg·kg^−1^. The negative control was divided in to two groups including animals treated orally with 1 mL of ricin oil or 0.2 mL of water using an intragastric tube. Test groups received the infusion or aqueous extracts at doses of 800 mg·kg^−1^ or the ethanolic extract at the dose of 400 mg·kg^−1^, resuspended in 0.2 mL of water. This assay was conducted for twenty-two days, after which the blood to be used for the evaluation of serum levels was collected from all animals. After different drug administration periods (no administration—T0, one dose—T1, repeated doses for 7 days—T2, and repeated doses for 14 days—T3), 1 mL of blood was collected by heart puncture, without anticoagulant substances, in order to obtain serum samples, and further analyzed according to several biochemical variables such as chloride, magnesium, phosphorus, and alanine aminotransferase by using commercial kits (Labtest Diagnostic S.A., Lagoa Santa-MG, Brazil). 

### 2.5. Histopathological Analysis

All animals that survived 22 days of treatment were submitted to laparotomy for removal of the small intestine and liver. These tissues were fixed in 10% neutral buffered formalin. The fixed samples were dehydrated in an ascending series of ethanol, cleared in methyl benzoate, and embedded in paraffin wax. Four-*μ*m-thick sections were obtained using a microtome and stained with hematoxylin and eosin. The photomicrographs were taken by digital camera (AxioCam MRc 5, Carl Zeiss) driven by software Axio Vision 4.6.3. (Carl Zeiss) coupled with an optical microscope (Carl Zeiss). Tissue lesion in the intestine was classified by the occurrence of villi alterations and ulceration or inflammatory process. Tissue lesion of the liver was determined by the presence of congestion, hydropic and fatty degeneration, inflammatory infiltrate, and necrosis. 

### 2.6. Statistical Analysis

Results were expressed as means ± SD of four separate experiments. Data from all assays were subjected to a Shapiro-Wilk test of normality statistical analysis of data obtained was performed using one-way analysis of variance (ANOVA), and the differences among group means were analyzed using Dunnett's multiple comparisons test. Analysis was performed using BioEstat software (v. 5.0). Values were held to be significant if *P* < .05. 

## 3. Results

### 3.1. Effect of Leaf Samples of *E. dysenterica* on Gastrointestinal Transit of Charcoal Meal

**Figure 1:**
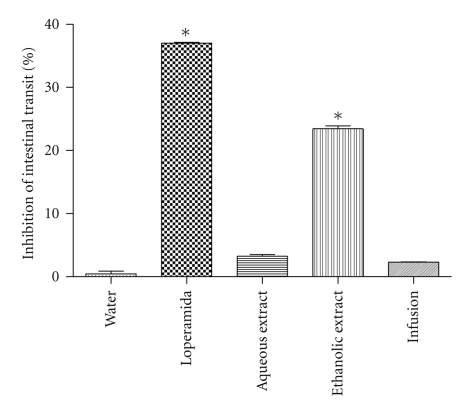
Evaluation of antimotility activity after administration of infusion and aqueous extract at the dose of 800 mg·kg^−1^ and ethanolic extract at the dose of 400 mg·kg^−1^. The positive control group received loperamide at the dose of 2 mg·kg^−1^, and the negative control group was given 1 mL of water orally. Vertical bars represent standard error. Asterisks indicate statistical difference from control (*P* ≤ .05) by Dunnett test.

Initially, experiments carried out on intestinal transit after charcoal meal administrations were conduced. The ethanolic extract at the dose of 400 mg·kg^−1^ and loperamide at the dose of 2 mg·kg^−1^significantly inhibited (*P* < .05) intestinal transit in rats by 24% and 37%, respectively (Figure 1). Nevertheless, aqueous extract and infusion did not cause any effect on intestinal transit, showing similar data to those of the negative control (water).

### 3.2. Evaluation of Ion Absorption after Ricin Oil-Induced Diarrhea

**Figure 2:**
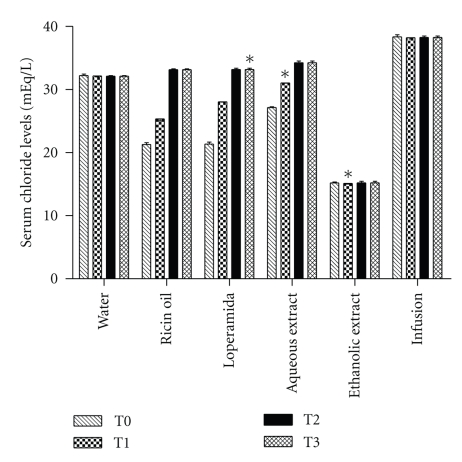
Analysis of the antisecretory effect of infusion, ethanolic and aqueous extract of *E. dysenterica* utilizing as parameters alterations in serum chloride levels at different periods: no treatments (T0), after one dose (T1), seven-day dose (T2) and 14-day dose (T3). Negative control group received 1 mL of water and ricin oil (10 mg·kg^−1^). Positive control group received loperamide (2 mg·kg^−1^) and experimental groups received infusion and aqueous extract at the dose of 800 mg·kg^−1^ and ethanolic extract at the dose of 400 mg·kg^−1^. Vertical bars represent standard error. Asterisks indicate statistical difference from control (*P* ≤ .05) by Dunnett test.

In the present study, diarrhea was induced with ricin oil, and the antidiarrheal that effect produced by samples was analyzed utilizing chloride, magnesium, and phosphorus levels at different periods. In animals treated with loperamide, levels of chloride increased by 41.2% (*P* < .05) (Figure 2). Animals treated with the aqueous and ethanolic extracts also showed a significant increase in levels of chloride, by 20.0% and 30.0%, respectively (*P* < .05). The other treatments showed no significant variation in serum levels of chloride with a consecutive dose of 800 mg·kg^−1^ for a period of 14 days (Figure 2).

**Figure 3:**
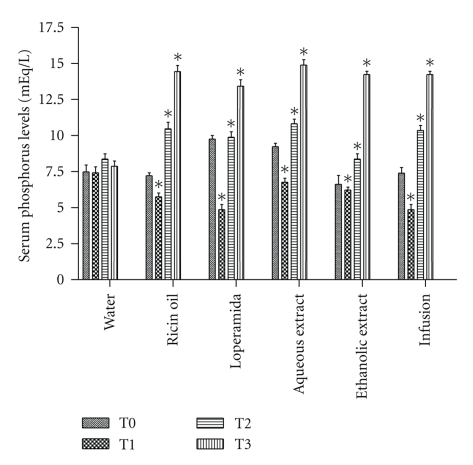
Analysis of antisecretory effect of infusion, ethanolic and aqueous extracts of *E. dysenterica* utilizing as parameters alterations in serum phosphorus levels at different periods: no treatments (T0), after one dose (T1), seven-day dose (T2), and 14-day dose (T3). Negative control group received 1 mL of water and ricin oil (10 mg·kg^−1^). Positive control group received loperamide (2 mg·kg^−1^), and experimental groups received infusion and aqueous extract at the dose of 800 mg·kg^−1^ and ethanolic extract at the dose of 400 mg·kg^−1^. Vertical bars represent standard error. Asterisks indicate statistical difference from control (*P* ≤ .05) by Dunnett test.

Analysis of phosphorus concentration showed initial decrease in animals' serum levels when treated with ricin oil (23%), loperamide (33%), aqueous extract (10%), ethanolic extract (18%), and infusion (33%) (Figure 3). Phosphorous in these groups decreased significantly (*P* < .05) after the administration of only one dose. Continued use could lead to hyperphosphatemia in animals treated with ricin oil (80.5%), loperamide (68.4%), aqueous extract (85.4%), ethanolic extract (75.7%), and infusion (75.7%) (*P* < .05) (Figure 3). 

**Figure 4:**
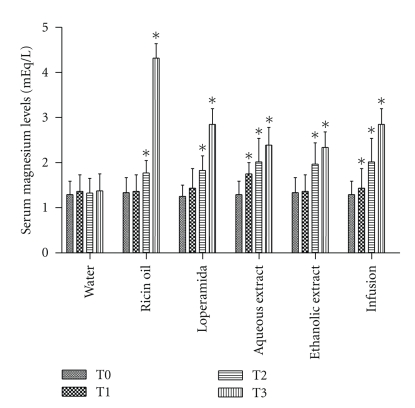
Analysis of antisecretory effect of infusion, ethanolic and aqueous extracts of *E. dysenterica* utilizing alterations in serum magnesium levels at different periods: no treatments (T0), after one dose (T1), seven-day dose (T2) as parameters, and 14-day dose (T3). Negative control group received 1 mL of water and ricin oil (10 mg·kg^−1^). Positive control group received loperamide (2 mg·kg^−1^), and experimental groups received infusion and aqueous extract at the dose of 800 mg·kg^−1^ and ethanolic extract at the dose of 400 mg·kg^−1^. Vertical bars represent standard error. Asterisks indicate statistical difference from control (*P* ≤ .05) by Dunnett test.

Magnesium level analyses of serum were altered in rats treated with ricin oil (165%), loperamide (82.8%), aqueous extract (58.8%), ethanolic extract (53%), and infusion (82.8%) (*P* < .05) (Figure 4). These alterations in serum magnesium levels were mainly observed after consecutive doses (T3), causing a clear increase of over 100%. Moreover, it is important to observe that in all analyses of serum levels, the animals that received 1 mL of water (negative control) did not show any modifications in the concentrations of this ion. 

### 3.3. Histopathological Analysis

**Figure 5:**
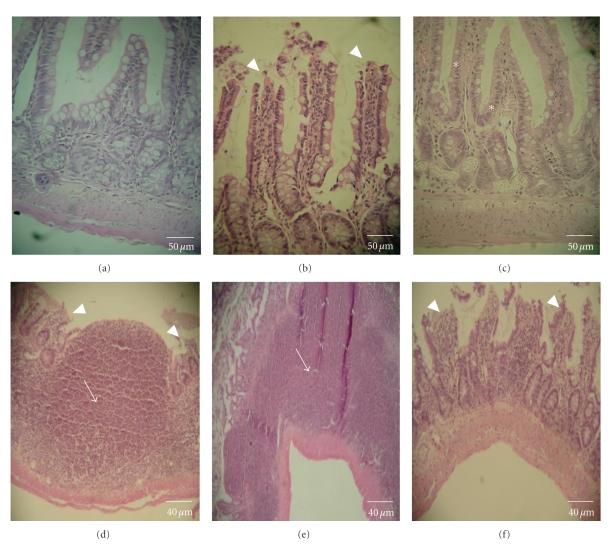
Small intestine sections of rats treated with water (a), infusion of leaves of *E. dysenterica *(b), ethanolic extract of leaves of *E. dysenterica *(c), aqueous extract of leaves of *E. dysenterica* (d), ricin oil (e) and loperamide (f). The arrows indicate intense mononuclear cell inflammatory infiltrate in the mucosa and submucosa; arrow tips indicate changes in villi morphology and the asterisk indicates congestion.

The histopathological analysis of the animals, after 22 days of administration of treatments, showed significant alterations in intestine and liver tissue. In general, the small intestine evidenced the presence of an inflammatory process causedtoall test groups. However, in the animals treated with infusion, it is important to note the presence of intense inflammatory infiltrate, partial loss of intestinal villi, and a complete mucosal alteration (Figure 5(b)). Animals that received ethanolic extract showed congestion and changes in the morphology of the villi (Figure 5(c)). Ulceration and intense inflammatory infiltrate were observed in animals treated with aqueous extract and ricin oil (Figures 5(d) and 5(e), resp.). Animals that received loperamide showed changes and destruction in the morphology of the villi (Figure 5(c)). Moreover, it should be noted that aqueous extract and ricin oil caused greater damage to the intestinal tissue than to the other analyzed groups. 

**Figure 6:**
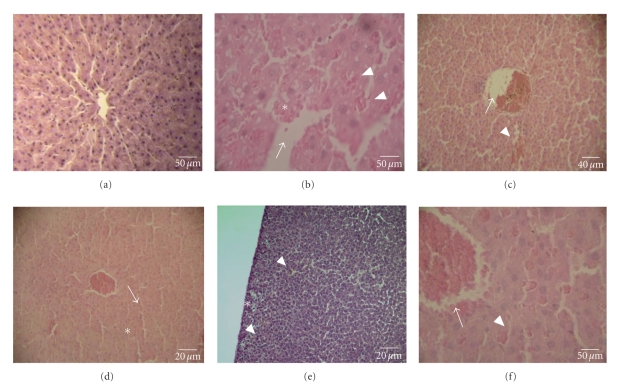
Liver sections of rats treated with water (a), infusion of leaves of *E. dysenterica *(b), ethanolic extract of leaves of *E. dysenterica *(c), aqueous extract of leaves of *E. dysenterica* (d), ricin oil (e) and loperamide (f), The arrows indicate congestion in the centrolobular vein, the arrow tips show congestion in the capillary sinusoids and the asterisks indicate hydropic degeneration in b and fatty degeneration in d. Figures b and f also show portal/periportal oedema formation. Figure e present numerous inflammatory process spots.

Histopathological analysis of the liver after infusion revealed that the sample caused congestion in the centrolobular veins, and this symptom could be associated with a congestion focus in the sinusoid capillary, hydropic degeneration, and portal/periportal oedema formation (Figure 6(b)). Similar data were observed in animals that received ricin oil, and a cluster of cords of hepatocytes was also described in association with this alteration, except for the presence of an inflammatory process and the absence of portal/periportal oedema formation in this group (Figure 6(e)). It should be emphasized that animals that received water did not show any modification (Figures 5(a) and 6(a)). Furthermore, the consecutive administration of loperamide caused the greatest liver alterations. Animals that received loperamide, ethanolic and aqueous extracts showed hydropic degeneration, sinusoidal congestion in the capillaries, and portal/periportal oedema formation (Figure 6(f)). 

### 3.4. Analysis of Serum Alanine Aminotransferase

**Figure 7:**
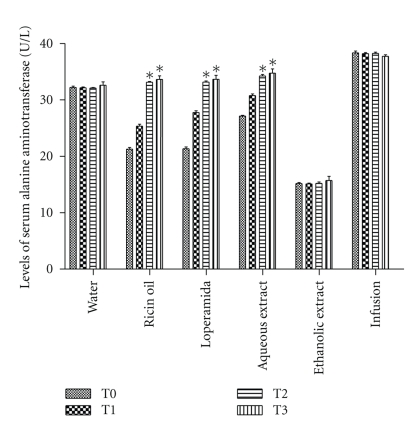
Evaluation of hepatotoxicity in rats after oral administration of infusion, ethanolic and aqueous extract of *E. dysenterica* utilizing as parameters alterations in the levels of serum (U/L) alanine aminotransferase (ALT) enzyme. Values of mean and standard deviation of the effect exerted by the samples at different periods: no treatments (T0), after one dose (T1), seven-day dose (T2) and 14-day dose (T3). Negative control group received 1 mL of water and ricin oil (10 mg·kg^−1^). Positive control group received loperamide (2 mg·kg^−1^) and experimental groups received infusion and aqueous extract at the dose of 800 mg·kg^−1^ and ethanolic extract at the dose of 400 mg·kg^−1^. Vertical bars represent standard error. Asterisks indicate statistical difference from control (*P* ≤ .05) by Dunnett test.

The levels of alanine aminotransferase (ALT) increased in animals treated with ricin oil (54%), loperamide (54%), and aqueous extract (26%) (Figure 7). It is noteworthy that despite the increase in enzyme serum levels and tissue damage observed in the histopathological analysis, none of the treatments exceeded the reference range for this rat's enzyme, which is 35.1 ± 13.3 U/L [19].

## 4. Discussion

Diarrhea is a consequence of innumerable pathologic conditions. Usually diarrhea is caused by altered motility and fluid accumulation in the intestine lumen, which can be occasioned by an increased secretion of electrolytes (secretory diarrhea), an enhanced ingestion of osmotic substances (osmotic diarrhea), or the presence of a virulent microorganism (infectious diarrhea) [3, 6, 24]. For this reason, in the present study, antidiarrheal properties of infusion, ethanolic and aqueous extracts of leaves of *E. dysenterica* were examined using various parameters, including intestinal motility and ionic absorption. 

It was shown that the ethanolic extract of leaves of *E. dysenterica *significantly inhibited intestinal transit in rats, while the other two extracts were unable to do that. Similar effects are described by Sagar and collaborators [19] with the use of *Lantana camara* methanolic extract against neostigmine as promotility agent. Khan and collaborators in [25] showed the antidiarrheal activity from Arque-Ajeeb, a mixture of *Mentha arvensis*, *Trachyspermum ammi,* and Kaphoor, which was able to inhibit intestinal transit in serotonin-induced diarrhea and PGE2-induced small intestine enteropooling. Various plant extracts are described as having antidiarrheal activity, such as aqueous extracts of *Guiera senegalensis* roots [26] and *Byrsocarpus coccineus *[27], and also methanol extracts of *Xylocarpus granatum* bark [12], *Asparagus pubescens *root [10], *Dalbergia lanceolaria* bark [13], and others. 

Antidiarrheal activity was found in plants possessing tannins, alkaloids, saponins, flavonoids, steroids, and terpenoids [28]. Abascal and Yarnell in [29] comment on the tendency to use plants for treatment of irritable bowel syndrome (IBS). These authors describe the use of bayberry (*Morella cerifera*) root bark, meadowsweet (*Filipendula ulmaria*) leaf and cranesbill (*Geranium maculatum*) root; these plants all produce tannins that bind the fluid in the colon, inhibiting the protective excretory function of diarrhea. Antidiarrheal activity is also described in the model of secretory diarrhea induced by cholera toxin, after ingestion of a mixture of SP-303 with procyanidin oligomers derived from the latex of *Croton lechleri*, known as Sangre de Drago [30]. 

The antidiarrheal activity of these compounds has been attributed to antimotility and antisecretory effects, and/or antimicrobial action [31]. In general, the presence of secretory diarrhea results in various electrolyte disorders. In the present study it was observed that the ethanolic extract of *E. dysenterica *causes electrolytic disturbance (affecting levels of chloride, magnesium, and phosphorus). Ion concentration analyses suggest that the antidiarrheal action was not caused by an antisecretory mechanism in the diarrhea model induced by ricin oil. It is known that ricin oil causes secretory diarrhea, since ricinoleic acid induces diarrhea through a hypersecretory response [32, 33]. Ricin oil reduces Na^+^ and K^+^ absorption and decreases Na^+^ and K^+^ ATPase activity. These actions, related to ricin oil's diarrheal effects, are involved in adenylate cyclase activation, secretion of mucosal cAMP, induction of platelet activating factor, increase in prostaglandin biosynthesis, and modification of nitric oxide concentration [12–34]. Although these numerous mechanisms have been proposed, the overall consensus is that ricin oil-induced diarrhea stimulates prostaglandin formation; consequently, a drug that decreases the synthesis of prostaglandins can be used in the treatment of diarrhea [14–34]. 

Kaysar and collaborators [35] reported that the lowest serum phosphate concentration is associated with laxative abuse, which occasions severe diarrhea, resembling that induced by ricin oil. This showed that after one dose, these samples probably have no antidiarrheal activity. Moreover, phosphorus concentration being affected by different factors, such as vitamin D overdoses and laxative abuse or enemas (which are also absorbed when given rectally), occasions severe diarrhea [36]. In general, when serum phosphorus levels decrease, reduction in urinary phosphorus excretion and stimulation of the 1-alpha hydroxylase enzyme in the kidneys were observed [37]. However, in the presence of hyperphosphatemia, as observed after prolonged use of all samples, including loperamide (positive control), a rapid increase in urinary excretion of phosphorus occurs, mediated by the serum phosphorus level, parathyroid hormone (PTH), and fibroblast growth factor 23 (FGF23) [38]. This probably explains why loperamide has no direct effect on absorption, since its action mechanism is related to interaction with the *μ*-opioid receptor, prolonging intestinal transit [39]. Abnormalities of chloride, phosphorus, and magnesium homeostasis are common and can be associated with dehydration caused by diarrhea [40]. Other authors report that hypomagnesemia involves intestinal loss, malabsorption, and abnormality of vitamin D and parathyroid hormone metabolism [41, 42]. 

**Figure 8:**
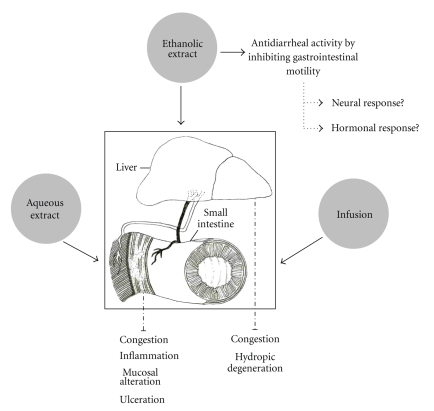
Hypothetical diagram summarizing the effect of ethanolic and aqueous extracts and infusion of *E. dysenterica* on the small intestine and liver.

Several antidiarrheal present toxicity effects, and for this reason, we make available the action of the ethanolic and aqueous extracts and infusion of *E. dysenterica* on the small intestine and liver, which is explained in Figure 8. The histopathological analysis of the small intestine showed serious injury to intestinal villi in animals treated with the infusion, ricin oil, and loperamide. The lesions in the epithelium observed in animals treated with these samples, including hemorrhage (data not shown), are indicative of several possible mucosal injuries. Some authors suggest that intestinal hemorrhage is associated with a significant localized inflammatory response in the mucosa and serosa, which they attributed to intestinal endotoxemia [43, 44]. The persistent evidence of inflammation in the colonic mucosa of animals treated with infusion, ricin oil, and loperamide showed that excessive diarrhea observed could be caused by changes in epithelial permeability. These modifications could be related to modifications in tight junction function, which are often observed in ulcerative colitis [45]. 

 Histopathological analysis of the liver in animals treated with loperamide, ethanolic and aqueous extracts showed hydropic degeneration, sinusoidal congestion in the capillaries and portal/periportal oedema formation. Similar results were described by Vasconcellos and collaborators [46], using leaves of *Capraria biflora.* It should be noted that the liver is a major site for the biotransformation, accumulation and excretion of exogenous chemicals, and that damage in the nearby tissue indicates possible toxicity [47].

The most common cause of abnormal liver enzymes in the sick patient is secondary liver changes that arise from a primary nonhepatic disease [48]. It is important to understand the reactive nature of the liver in response to other disease conditions. In such reactive situations, the hepatopathy is not the primary hepatic disease [49], and as we have seen in this study, there is a positive correlation between liver damage and changes in serum ALT. 

An interesting observation, which also causes concern, is that although the infusion showed no biological activity in the tests used here, it did, however, present severe damage to the intestinal tissue and liver. These findings are worrying, since the Cerrado population frequently uses tea made from *E. dysenterica* leaves in the treatment of diarrhea.

In summary, data reported here demonstrated antidiarrheal activity by inhibiting gastrointestinal motility in animal models. Due to these findings, we believe that *E. dysenterica* could be a potential source of a new antidiarrheal drug, despite the above-mentioned toxicological effects. Results presented here confirm the popular use of *E. dysenterica* and its well known medical properties in folklore medicines. These data are extremely important, since the use of medicinal plants has recently increased in developed countries because of disillusionment with traditional medicine. In developing countries, the strategy of alternative medicine continues to be used due to low costs and abundant availability. The contribution of traditional knowledge can improve the development of more effective drugs with minimal or no side effects, also helping to conserve biological resources.

## Abbreviations

5-HT4: 5-hydroxytryptamine (serotonin) receptor 4
ALT: Alanine aminotransferase
DMF: Dimethylformamide
FGF23: Fibroblast growth factor 23
HPLC: High-performance liquid chromatography
IBS: Irrifigure bowel syndrome
mEq/L: Milliequivalents per liter
PTH: Parathyroid hormone
T0: No administration
T1: One dose administration
T2: Consecutive dose for seven days
T3: Repeated dose for fourteen days
TFA: Trifluoroacetic acid
WHO: World Health Organization.
